# Supplementary material for: Perceptual clustering in auditory streaming
Source: PLoS Comput Biol. 2025 Jul 11;21(7):e1013189. doi: 10.1371/journal.pcbi.1013189 (PMC12273984; doi:10.1371/journal.pcbi.1013189)
Supplement: Attachment — Submitted filename: Reviewer Response PLoS Comp Bio subm 2024 .pdf [file pcbi.1013189.s002.pdf]

Response to reviewers

We are grateful for the considerate comments by the reviewers. We have implemented all suggested changes and the manuscript has clearly been improved by these. Below we go through the reviewers' comments, marked in *italic.*

Reviewer #1

This is a nice paper on a model of causal inference that sensibly extends to stimuli with more than two items. The original CI model from Koerding and colleagues was based on only a pair of stimuli and a generative model with only two scenaries (same-source and separate-sources). As the authors know, I and many others have published reams of papers based on the dichotomous setup. In fact, I have toyed with a model for scenarios with more than two items (i.e., multiple flashes and beeps), and the combinatorics made for very clunky modeling (and I've never finished nor published the project). So, this straightforward bit of modeling is a welcome addition. I have a bunch of comments, but all in all I very much like this paper.

We are very grateful for the appreciative comments by the reviewer!

Specifics:

** Eq. 1: Some variations and clarifications here are worth a mention. Delta-t here is ISI, and I imagine that ISI and SOA might both matter, but that gets into the complexities of rhythm perception, and the experiments here all avoid any need for that, so it's fine as is. Delta-f is never clearly defined. One might imagine f is in Hz, but all Methods describes them in log scale (multiples of a semitone), so you might say that this is a Gaussian in something like "log-frequency velocity". I only know superficial things about the stream-segregation literature. Is log-frequency difference the right variable here (independent of base frequency)? Does stream segregation also take into account the circular nature of pitch perception, so that octave leaps as LESS likely to segregate than small leaps?*

Delta-f is indeed on a log-frequency scale. We have added this precision after Eq. 1 (L111). As the perception of sounds is logarithmic, it makes sense from a phenomenological point of view to use this scale; and this is how it has been done traditionally in the stream segregation literature. We do not know of papers exploring if the circularity of pitch perception does tend to reduce segregation of tones. Although this is a very interesting point, our Experiment 1 contradicts the notion: an octave being equal to 12 semitones, Figure 6 would display a reduction of the stream segregation effect at 12 semitones should it be the case.

** 122-145: I had some trouble plowing through this section to get a clear handle on what was being assumed and whether it justified all the simplifications here. A clear generative model*

would have been helpful (possibly with a model diagram). When you apply Bayes Rule to obtain the 2nd line of Eq. 5, you make some kind of conditional-independence assumption. You also say that just above Eq. 5, but it's unclear exactly what is conditionally independent of what, nor how strong (and acceptable) an assumption that might be. True, the denominator in that 2nd line can be figured out as whatever normalizing constant needed. In any case, some clarification along the way would be helpful. Note that in the first and 2nd lines of Eq. 5 you have "S_2,S_3" just after a condition "|", when in fact you mean "S_1,S_2".

We acknowledge that our description of the derivation of the Bayesian inference process could have been clearer. We have created a graphical representation in the Supplementary material (shown below). If the reviewer finds it useful we are happy to move it to the main text.
We have also for clarity rewritten the derivation in Eq. 5.

Thank you for pointing out the error regarding numbering in Eq 5, which we have now corrected.

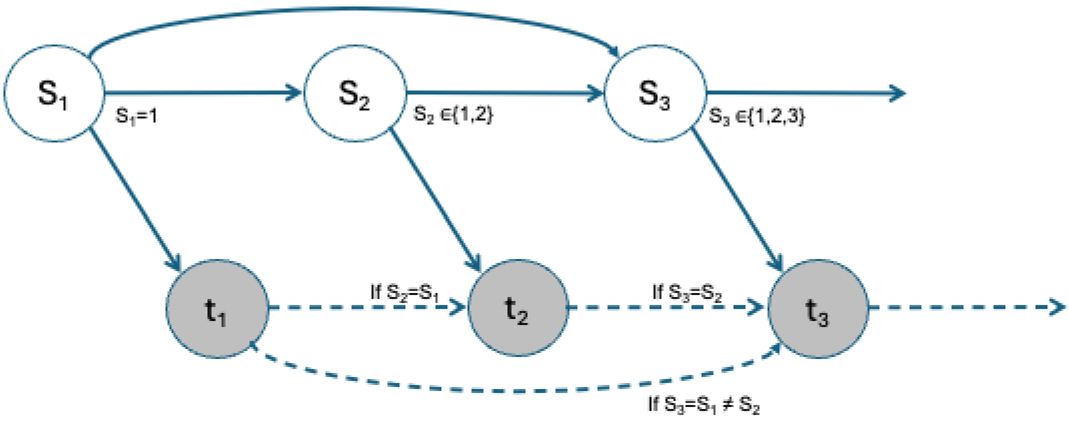

Figure for graphical model: Graphical representation of the main model. Sources (S) generate observed tones (t). First source is always indexed as 1, subsequent sources can either be same or different, with probabilities generated according to the Chinese Restaurant process (see main text Eqs.2-3). The properties of each tone (frequency, on-time, off-time) are conditional upon the previous tone with the same source. For more details see the main text.

** Figure 6: You should label the color bar in (a) (yes, it's in the legend, but it's nice to be able to look at a figure and understand it as is).*

The figure has been changed.

** 288: I'd avoid obscure acronyms like "ASA".*

We have replaced the acronym by its meaning, Auditory Scene Analysis.

** 300: "If medium tones were to be part...". I totally tripped over this sentence and couldn't understand why UNTIL I noticed the obscure text at the top of Figure 9 that talked about the 22 and 11 repetitions. This repetition is never described in the running text and even less clear, but important, is that during the repetitions, the SOA from M1 to M2 and the SOA from M2 to the next repetition's M1 are identical. That's why an inversion is hard to detect. Of course, potentially the participant could discriminate the two sequences by noting which tone came last, but they didn't know it was an integer number of repeats. So, you should clarify all of this somewhere.*

Thank you for your remark. We have used your suggestion to add more details right after your original point of confusion and we hope this will clarify things for future readers (L329-333). Regarding the possible note of which tone came last, this was mitigated by a general fade in and fade out of the melodies. Therefore, melodies sound like they are part of a continuous chain within which there is no real starting or ending point.

** Table 1: The labels of the rows as "Different" and "Similar" is unclear. What you mean is that these are rows for stimuli in which the two sequences were identical vs. inverted. Maybe you should label the rows as Hit Rate and False-Alarm Rate and clarify in the legend.*

Thank you, it was indeed unclear. We have implemented your suggested change.

** Figure 8 and text above: Yes, it's pretty disconcerting that ELBO was at chance or very close to it for about half your subjects (and all models including yours). The figure should repeat the key for the colors from Figure 6.*

Indeed, we found that 6 of our subjects were at chance level reflecting the difficulty of the task. We appreciate the suggested change to the figure and have modified it accordingly.

** Discussion: This is still a very fancy model to computer and one wonders whether the brain perhaps does something more heuristic and simpler. Perhaps some kind of greedy algorithm that collects evidence and keeps around only a single interpretation or a very small number, plus confidence, accumulating evidence without keeping lots of hypotheses around.*

This is a very valid point. We have now included a short discussion on this in the Discussion (L444-452)

** Eq. 8: You are using 1 and 0 to represent responses of 1 and 2, which seems unnecessarily confusing.*

This was indeed unnecessarily confusing. Reviewer 3 had a similar remark, and we corrected r_k to reflect responses as 1 or 2.

** 494: The alternative to a single-stream hypothesis does not have to be half-half.*

Yes, the sentence has been changed (L553).

** Eq. 11 and surrounding text: This supports the 2-stream response even if there's a high probability of 3 or more streams.*

Indeed. This is to reflect the possible responses from the experimental setting: in Exp 1, participants can only respond with 1 or 2.
It can be suspected that if a participant perceived more than 2 streams, they would also probably respond with a button press of 2, i.e. that they do not experience a single stream.
We have now tried to make this clearer in the manuscript (L.554-556).

Reviewer #2

The authors develop and test a novel Bayesian model that infers the causal structure of sound sources in auditory streams. Specifically, they reformulate auditory stream segregation as a Bayesian clustering problem: They develop a Bayesian model with a non-parametric prior based on a Chinese restaurant process (CRP) that allows updating of posterior distributions over potential causal structures with potentially infinite number of sources, only inferred from previous sensory inputs.
Overall, by assuming a CRP, the model elegantly infers causal structure of complex sound sources, going beyond previous causal inference models that only take two potential causal structures into account. The model qualitatively replicates important effects in auditory stream segregation (e.g. effects of tone frequency and ISI and the Gallopping effect), and is quantitatively fitted to a replication experiment of the Gallopping effect and a novel experiment testing a novel model prediction (i.e., the effect of melody segmentation on melody discrimination). The manuscript is well written and comprises an important novel modelling approach for a fundamental problem in auditory perception (causal inference on complex sound source structures in auditory streams). Importantly, the CRP model could be generalized to perceptual problems of causal inferences in other sensory modalities and specifically multisensory perception, so the work is very innovative and has wide applicability as well as relevance. Yet, I have some comments and concerns on the modelling approach, the experimental data and the presentation of results:

We appreciate the positive comments by the reviewer.

Intro
• L. 107 “the probability”, this definition requires further explication: Which probability is meant here exactly, I assume a likelihood for tone frequency and temporal distance, conditioned on the

same source as the previous tone? The argument that the frequency of a tone from the same source cannot change instantly is plausible (and therefore frequency distance decreases the probability), but why does "the probability" increase with a longer ISI between previous tone offset and onset of the current tone? This counterintuitive assumption is presumably need to model the effect of presentation speed in Fig. 3a/b, but the rationale is unclear. From natural sound statistics, I would assume the reverse: longer pauses between two tones make it less likely that they have the same source (i.e., a new source is more likely).

The paragraph indeed has the goal to explain the likelihood. However we explain it in terms of the generative (forward) model, and therefore prefer to use the term probability in order to avoid any confusion with the likelihood function, which performs the 'inverse' inference.
We have now tried to clarify the goal of the paragraph.

Sound production (as well as perception) clearly operates on multiple timescales. What we are arguing in the motivation for the model is that on a short time scale (milliseconds) a physical system will need time to change configuration so as to produce a different frequency and that the time to make that change depends on difference in tone produced. If a tone of the same frequency is to be generated no change in the system is required, while a larger tone difference requires more time. The reviewer is of course right that on longer time scales (seconds) other processes happen, causing a new source to be more likely after a long gap. This could be added as an extra term for the generative process for the successive sources. We have added a note to this effect and rephrased the paragraph to try to make this more clear.

• L. 110: "We here assume that the observer has a perfect noise free access to the generated auditory frequencies." This is not a realistic assumption in any sensory system, how would the model be complicated by adding sensory noise? Or more generally, a hallmark of Bayesian models of perception is that they take sensory uncertainty into account, which effects would the model predict for e.g. low vs. high sensory noise in frequency and time estimation?

The reviewer brings up an interesting discussion point. In the general case any perceptual signal received by the observer will be inherently noisy/variable, although in the case of the stimuli we use the sensory noise is much smaller than any structural uncertainty in the task (more on this below, when responding to the same reviewer's comment in Discussion).
Intuitively it would be expected that adding further sensory noise, apart from increasing response variability, would lead to a tendency to decrease the number of perceived streams (as the added uncertainty makes it harder to reject the hypothesis that the stimuli originated from more than one stream) however more work on the model would have to be done to confirm thes (see also below).

• Equation (2): The alpha parameter is undefined, I assumed it is meant as a small probability? In general, the Chinese restaurant process / Dirichlet process could be explained to increase readability in the Introduction.

The alpha parameter is indeed fitted as part of the parameter fitting. We now make this clear when discussing equation 2.

We have clarified in the Introduction that we are indeed referring to the Chinese restaurant process (L27-29).

• Fig. 2 and equation (6): The likelihood for a tone from a new source is highest when it is closed to f0, i.e. the average or midpoint of frequency of previous tones in a trial. This definition of f0 is counterintuitive to me: I would rather expect that a new source is likely if the frequency is very different from previous frequencies (i.e., higher or lower). Clustering of frequencies around a mean/midpoint is rather likely if the same source emits more tones.

The frequency of a tone from a new source is essentially drawn from a prior over frequencies, i.e. prior to receiving any information a tone from a new source is expected to be drawn from the prior distribution. In our case we assume a prior distribution centered on the range of frequencies displayed in the experiment. We have now clarified this in the manuscript (L182-184).
Note also that during inference the likelihood function will already favor tones from new sources to be different from previous tones, counteracting any central clustering.

Results, Exp. 1 / 2:
• Fig. 6 gives a somewhat incomplete impression of the behavioral and modelling results: Why is the model prediction only shown for subject 8? Why is not the average data shown for 15 participants but only 7 selected participants? Showing mean data and mean model predictions for all participants (or behahavioral data / model predictions for several subjects side-by-side) would give a more comprehensive impression. What is the overall model fit of the CRP model (e.g. R^2) for Exp. 1 and 2?

There is considerable variability in the behaviour of individual subjects, hence we found it more useful to present the individual subject data, as opposed to average over the data. Furthermore the parameter fitting is on individual subject data, hence presenting fits to individual subjects is also more useful than averaging over all data.
We now present both data and fits from four subjects, and have put data and model fits from all 15 subjects in Supplementary materials.

As the responses are binary in Exp 1 and 2 it would not be appropriate to use R2. While it is possible to instead calculate the correlation across conditions (r=0.945, R^2=0.893) this would not reflect the full variability of the data. We are more comfortable using the measure of ELBO, which can provide an upper (perfect fit) and lower bound (purely random response). In Fig. 6c we already show these upper and lower limits. If we were to convert these into a proportion (ELBOmin-ELBO)/ELBOmin, the average ELBO proportion would be 0.419 for Exp.1 and 0.127 for Exp 2, which has the advantage that it can be interpreted the same way as an R2 value. We have made a note of that in the legend of the figures for Exp 1 and 2.

• The main CRP model is compared to competing models with simpler ad-hoc priors in Exp. 1/2. However, how does the CRP model compare to models which use different non-optimal/heuristic decision strategies, such as stochastic sampling of same source / new source for each tone or deciding for same / new source based on a fixed threshold on pitch increment and ISI (cf. Acerbi et al., 2018 Plos Comp Biol)?

We have in previous perceptual modeling studies examined the use of different decision strategies, e.g. mean of posterior, max of posterior, or sampling (probability matching). This is an interesting research area to pursue for continuous responses, see e.g. Wozny, Beierholm & Shams 2010, as well as Sanborn & Beierholm 2016.
In the current study, given a set of stimuli the model generates a posterior probability for each of the possible tone-to-source assignments (e.g. (S1=1, S2=2, S3=1…,)), and creates a single binary response. As an optimal response we assume participants sum (marginalise) across the posterior probabilities of different tone configurations, arriving at the binary response (1 or 2 streams, same or different). But there are indeed other decision mechanisms possible, although they are less useful for this type of model.
Sampling from the posterior would not lead to any different results as the response is binary and the model is evaluated based on a softmax over multiple trials.
Any subjects using a *fixed threshold* in either pitch increment or ISI would be easy to detect in the data in Exp 1 as they would show up as vertical or horizontal lines/boundaries in FIg. 6b.
We did also (ad-hoc) examine the *maximum of the posterior* (much harder to fit parameters over given the discontinuous parameter-to-probability mapping) and found that the maximum corresponded to the typical response of the model with marginalised posterior. This is mentioned in the Discussion (third paragraph) but we now more explicitly mention other potential strategies (L425-429).

• Exp. 2: The definitions of frequencies such as 3-3 (L. 326) is unclear at this point of the manuscript, frequency difference between low-middle, or middle-high tones? It is defined in the methods, but a brief definition here would help the reader. Even though the predictions for Exp. 2 are nicely explained, it remains underexplained how this translates into d prime differences. I.e., the readability for exp. 2 could be enhanced.

We have rewritten the paragraph to make it clearer what the experimental conditions were, and the transformation into d prime (L335-340 and 359-362).

Discussion

• The authors discuss the effect that later tones can retroactively change the causal attribution of previous tones. This is actually a very interesting consequence of causal inference from accumulating evidence, and could be more broadly discussed in the context of postdiction in perception (e.g., Choi & Scholl 2006; Stiles, Tanguay, Shimojo, 2021).

That is a very good suggestion and we have added a mention in the discussion on this (L439-443).

• The model accounts for 'causal uncertainty' about the generating structure. As mentioned above, further uncertainty from sensory noise is a more realistic assumption. How could this be incorporated into the model, and how would it change model behaviour (e.g. prediction of novel effects?)? The authors claim that the model is very general, e.g. "the framework allows more complex cues from audition and other modalities to be used as long as their perceptual difference can be quantified" (L. 89): I imagine that e.g. adding visual cues with uncertain temporal structure and integration of audiovisual cues based on relative precision could critically depend on modelling sensory noise. In other words, while mere clustering of stimuli into a Gestalt may neglect sensory noise, it becomes crucial when stimuli are not only grouped, but integrated or unified into a single percept.

We are grateful for the chance to comment on this issue, as it is indeed very relevant.
In this study we assume no perceptual noise, but assume variability in how stimuli change over time (tones can go up and down) providing a structural/causal uncertainty. In contrast, in our perceptual studies (Koerding et al 2007, Shams & Beierholm 2010) the causal structure is simple (either same or different sources) and noise-free, but these models include perceptual noise. Hence the studies assume noise/variability at different stages of the generative process. We are currently working on incorporating the non-parametric process into our previous models of causal uncertainty for multi-sensory perception, where indeed the presence of noise is crucial for performing integration. We have successfully modeled the behaviour of subjects in a previously published tri-modal study (Wozny, Beierholm, Shams 2008) using a simplified version of the Chinese Restaurant Process adapted to just three stimuli (unpublished) and are currently developing novel experiments to test these ideas further.
We have added a short mention of these ideas to the Discussion section (L497-501).

• For an example of causal inference / clustering in the visual modality, the authors may also discuss causal inference for visual motion perception (e.g., Shivkumar, DeAngelis, Haefner 2023)

Thank you, that is a very relevant contribution. We have included it in the paper (L528-529).

Methods

• The methods do not explain how the model predictions for replicating previous auditory stream segregation effects (Fig. 3/4) were generated.

All details for modeling the previous experiments (Fig. 3-5) are available in the legends of the respective figures. If we have somehow missed any details we are happy to provide them.

• For the sake of completeness/reporting standards, the authors may report some basic demographics (age, sex distribution) of the sample (if available).

The demographics were not a point of interest for us in this study hence age and sex were not recorded. For both experiments, all participants were under-graduate students at a Psychology programme (except for one of the authors included in Exp. 1) and received credits for their participation, making the demographics equivalent to similar studies.

• *Exp. 2:*

o Why was the different sequences presented in twice as many trials as the identical sequences, and not with equal proportion? This could have introduced a response bias towards a "different" response.

Thank you for this insightful remark. The decision to present different sequences in twice as many trials as identical sequences had two goals: first, to minimize the overall duration of the experiment, which was already lengthy and very demanding to participants. Additionally, with differences of 9+ between sequences, it becomes increasingly difficult to perceptually cluster tones, which may lead participants to in fact perceive fewer differences despite the imbalance in trial proportions. Theoretically, it could be argued to instead introduce the risk of the opposite bias : one towards "similar" responses, as participants might phenomenologically experience a greater prevalence of similar melodies despite the opposite imbalance. Supporting this, if anything, results from Table 1 could indicate a potential very slight bias towards "similar" responses: for example, in the 3-3 condition—considered relatively easy—approximately 27% of different trials were incorrectly identified as similar, compared to ~20% of similar trials misidentified as different. But importantly, as our analyses rely on differences between D-prime scores rather than absolute response rates, this imbalance is unlikely to meaningfully affect the results unless extreme ceiling or floor effects are present, which is not apparent in our data.

o Participants also provided confidence ratings in each trial. Did this data reveal any meaningful pattern regarding auditory stream segregation / the modelling approach?

Confidence ratings were originally included as an exploratory variable, as we did not have a priori hypotheses regarding their role in auditory stream segregation or the modeling approach. Preliminary analyses indicated that confidence ratings were essentially a much noisier measure of the participants' decision, offering no additional meaningful insights. As a result, they were excluded from the manuscript. We have added this missing information (L677-679).

• *Parameter recovery could be accompanied by model recovery for the CRP and competing models (relevant esp. for a novel model; cf. Wilson & Collins 2019 eLife).*

Model recovery can indeed be very useful, especially for comparing new against existing well-established models of a phenomenon. In our case our model is not only new, but does not have any existing probabilistic competitors. We have attempted in the manuscript to include

variants of the model that effectively work as competitors, most of which limit the standard model or operate on a restricted parameter set (e.g. alternative model C is a special case of model B where the prior probability is fixed at 0.5). As such we believe model recovery in this paper would be of limited use.
We are hoping that in the future more competitive models will be developed, and we have provided our source code, and are happy to provide help in performing competitive model comparison and model recovery.

The authors make all data and code available on OSF.

Reviewer #3

This paper brings an existing mathematical tool (nonparametric Bayesian clustering) to bear on the problem of modeling causal inference in the brain when there exist a potentially unlimited number of sources, focusing specifically on the case of auditory streaming. The key insight is that an existing peg fits an existing hole, and indeed it does. Most importantly, the authors do substantial legwork to demonstrate the quality of the fit by showing that this mathematical formalism naturally accounts for a range of existing (and one new) auditory psychophysics results, and that it can easily be fit to individual data, outperforming several alternative models. The goal of the paper is successfully accomplished, and the insight is interesting enough to merit publication.

One minor qualm I have with the argument here is that even though the generative model can now account for unlimited sources, we have only kicked the can down the road -- we still need a posterior approximation that makes the computations tractable. These approximations are not just mathematical conveniences -- they correspond to real strategies that the brain might employ to utilize limited resources, and they make behavioral predictions. This point need not be fully explored in this paper, but it deserves mention in the main body of the text and not relegation to the appendix.

This is part of the larger question of mechanistic implementation of the algorithm by the wetware, which the authors should acknowledge is left for future work. When the authors do mention mechanistic models, it is only for the purpose of evaluating them in competition with the current model. They should make it clear that their model is in want of a mechanistic implementation, and when appropriate should evaluate whether features of existing mechanistic models might help to fill this void. Mentioning Marr's Levels is not mandatory but could be useful.

I am a firm believer in devoting text in modeling papers to intuition-building. When the authors list perceptual phenomena accounted for by their model, I would like to see them offer some intuitive explanation for why their approach accounts for these observations. For me this was

particularly an issue in the discussions of stream segregation following the accumulation of sound pattern repetitions (starting on line 197) and the bouncing percept (213), key strengths of the model. Please explain why the model does these things!

Thank you for this great suggestion. We added observations for each qualitative comparison to build more intuition (L219-223; L233-239; L247-253).

Other minor complaints:
- The Galloping Effect section is strange after the section on Time and Frequency which already explained and invoked the galloping effect. Maybe there's a better way to organize this material?

This section was indeed a bit awkward. It has been removed and integrated into the “Time and Frequency” section.

- L12: "Inferring the generative model" is awkward language. Usually one would say "inferring the parameters of the generative model," though I see why this might not make sense for a "nonparametric" model.

We have changed our wording to “inferring which generative structure produced the observed stimuli” to make our sentence less ambiguous and consistent with the wording of Figure 1

- The "3-3" notation is used extensively in the presentation of Experiment 2 but is not well explained in the body text.

Thank you for this comment. It was indeed difficult to understand for readers, and we have therefore added an explanation (L335-340).

- L34: should be "renowned"
- Inappropriate commas on lines 16, 29, 31
- "i.e." should be "e.g." on line 16
- Missing comma on 353

Thank you, we have corrected those lines accordingly.

- L117: awkward notation -- why is n not indexed with the candidate source j? I think maybe this should say:
If the number of tones previously assigned to source s prior to event i is given by n_s^i = /sum_{k=1}^{i-1} delta(s - S_k) , then
p(S_i = s | ...
[etc... or you could leave out the equation for n_s^i since it's obvious]

We agree that the notation could have been clearer, and have now simplified it.

- L491 and below: is r_k drawn from {0,1} or {1,2}?

Thank you for your remark. Reviewer 1 had a similar one, and we changed Equation 8 & 9 to reflect that r_k is indeed drawn from {1, 2}.

- L498 -- I would find it helpful here to specify which parameters were fit for each model (one of which is beta, which you just introduced) -- I think this only becomes fully clear in the Supporting Information.

The model parameters were specified earlier in the text (Inference section L185-188), but for clarity we now also mention them in the Methods section (L559-561)
