# Supplementary material for: Perceptual clustering in auditory streaming
Source: PLoS Comput Biol. 2025 Jul 11;21(7):e1013189. doi: 10.1371/journal.pcbi.1013189 (PMC12273984; doi:10.1371/journal.pcbi.1013189)
Supplement: Attachment — Submitted filename: Response to reviewers for R2 for Non par paper in PLoS CB.docx [file pcbi.1013189.s003.docx]

# Response to reviewers for manuscript PCOMPBIOL-D-24-01574R1

We are again grateful for the comments by the reviewers which have improved the manuscript.

The reviewer comments are below in *blue italic*, followed by our responses.

*Reviewer #1: Re-review of "Perceptual clustering in auditory streaming"
Reviewer: Michael Landy*

*I liked this paper the last time, and I still do. Not a lot of comments:

Line 164: Isn't this simply another way of specifying \alpha (from Eq. 2)? Why isn't \alpha mentioned here?*

The reviewer is right that it is appropriate to mention \alpha here. We have modified the paragraph accordingly:

“The value of p(S_1=1,S_2=1) for an individual can be determined from fitting their data, and the value p(S_1=1,S_2=2) is simply 1– p(S_1=1,S_2=1). **Note also that due to Eq. 3, and that by definition p(S_1=1)=1, we can write p(S_1=1,S_2=2)=p(S_2=2|S_1=1)p(S_1=1)=\alpha/(1+\alpha).”**

*170: This text should refer back to Eq. 1*

Thank you, done

*You use \sigma in Eq. 1, where it is in units of semitones/sec, and again in Eq. 6, where it's in units of semitones. These can't be the same \sigma, and yet you act like you have only a single \sigma parameter. Clarify. In line 310 it says it's the former (semitones/sec).*

The reviewer is correct and we are grateful for them to notice this detail. The variance for the likelihood in the new-stream likelihood is indeed different. In practice, in the code for Eq. 6 we just assume an arbitrarily large variance value, as our modeling indicates it has little practical effect, is partially confounded with the CRP prior, and it reduces the model by one potential parameter. We have now changed the variable name to \theta and clarified that in the manuscript.

**“For the variance of this likelihood, \theta, we have set it to be an arbitrarily large value, to reduce the number of variables in the model and as it plays a limited role in practice.”**

*Fig. 2: Don't you need to specify that \alpha is forced to be less than one (to keep the new cluster with the smallest prior probability)?*

\alpha does in fact *not* need to be less than one, but is obviously required to be larger than zero. If it is larger than one it just influences the prior probability of assigning the second tone as one source to be less than 0.5, i.e. the second tone is more likely to start a second cluster.

As an example, assume e.g. that \alpha = 2. After the first tone (which has to be assigned to cluster 1) we can calculate for the second tone:

P(S_2_=1|S_1_=1)=ns/(i-1+alpha)=	1/(1+2)=1/3

P(S_2_=2|S_1_=1)=alpha/(i-1+alpha)= 2/(1+2)=2/3

But for the 11th tone, if all previous 10 tones were in cluster 1

P(S_11_=1|S_1:10_=1)=10/(10+2)=5/6

P(S_11_=2|S_1:10_=1)=2/(1+2)=1/6

Hence having \alpha larger than one is not a problem. We now state that we require \alpha>0 in the fitting.

*196: a verb is missing here*

The sentence should have been better written. We now state

“A well-known basic stream segregation phenomena (e.g. [11]) shows that increasing the speed at which auditory tones **are presented**, increases the probability that tones are perceived as coming from separate streams.”

*Fig. 3: These dendrograms don't really cluster the tones exactly as one would wish. In (b) one would expect 2/4/6 to be grouped. In (c) it should be 123 and 456. Thoughts?*

The reviewer raises a good point. The model does a good job at explaining the grouping at the first level (first two clusterings) as seen in both Fig 3b and 3c.

For 3c we would argue that it still does a reasonable job at the second level, emphasizing the relationship between the low tones (1, 3, 4, 6 …) vs high tones (2, 5, 8…).

While the dendogram for 3b low tones (blue) are fine (1,3,5)(7,9,11) for the high tones it seems to prefer (2,4)(6, 8,10)(12) as opposed to (2,4,6)(8,10,12).

This may indeed seem surprising, but a) it is at a secondary level of clustering, and thus less likely to be intuitive and b) it may be specific to the (arbitrary) parameters we had chosen. Different parameter values of e.g. the sigma parameter can lead to different clusterings at the secondary/lower levels of the dendogram:

*289: The supplement does NOT have the individual subject data and fits.*

We sincerely apologise, in the final version of the LaTex document the figure was lost. We have ensured (and double checked) that it is included now.

*Reviewer #2: The authors address most of my concerns very well, expect for the model recovery. Overall, the manuscript has improved, especially in readability, and the paper is an important contribution.
My point-by-point responses:
• Definition of probability of a common source: I agree with the authors that the probability for a common source depends on temporal distance between two tones on different time scales. I still the explanation in the manuscript too short, while the explanation in the response latter is clearer: If a sound source continues oscillating at the same frequency, two consecutive tones of the different frequency are unlikely while two tones (at the same frequency) with larger temporal distance are likely. I still find it difficult to follow their argument, maybe they could explain a bit more elaborately in the manuscript. An option would also be to plot the normal distribution (equ. 1) to give the reader a more intuitive understanding (e.g. supplemental).*

As suggested we have expanded the explanation, using partly text from our previous response:

“If two sequential sounds with the frequencies $f_{i-1}$ and $f_{i}$ are produced by the same source, $S_i=S_{i-1}$, the pitch cannot change at an infinitely fast rate: to make an oscillator change its frequency discontinuously would require an infinite impulse of energy.

**A physical system will need time to change configuration so as to produce a different frequency and that the time to make that change depends on the difference in tone produced. If a tone of the same frequency is to be generated, no change in the system is required, while a larger tone difference requires more time.”**

We already plot the normal distribution of the likelihood in Figure 2. We have now moved it up and refer to it earlier to help the reader.

*• Effect of adding sensory noise:
• Alpha parameter and Chinese restaurant process: These concepts have now been clarified, even though the CRP could be intuitively (very briefly) explained in the Intro to increase readability.*

We have added a very brief description of the process.

“ Intuitively, the algorithm assigns customers (data points) at a fictional restaurant to different tables (groups and clusters) based on the number of customers already seated at the table. As the number of customers assigned to a table grows, it becomes more likely to be assigned further customers, making it less likely that new customers will be assigned a new table.”

*• I agree that for a prior distribution for a new source assumes that the tone will still be in the range of previous frequencies (as a prior guess).
• Fig. 6/7: The authors now show model comparison results of the whole sample (with clear support for the CRP model in each participant in Exp.1 and most participants in Exp.2), a measure of overall model fit, and model predictions for more participants in the main text. The authors promise to show model fits from all participants for Exp. 1 in the supplemental, but unfortunately I do not find it there (only the graphical model and parameter recovery)??*

We sincerely apologise, in the final version of the LaTex document the figure was lost. We have ensured (and double checked) that it is included now.

*• Alternative decision strategies: The authors now discuss the possibilities of alternative decision strategies, leaving it to future research to investigate this, which is fine. One point: “Any subjects using a fixed threshold in either pitch increment or ISI would be easy to detect in the data in Exp 1 as they would show up as vertical or horizontal lines/boundaries in FIg. 6b.” 6B shows the ELBO values, I guess the authors refer to 6A (and in the promised supplemental figures), where indeed a fixed-threshold strategy would show as a clear horizontal/vertical pattern.*

Yes, our response referred to the previous version of the figure. The previous Fig 6b is now part of Fig 6a.

*• The authors now sufficiently explain the definitions of frequencies in Exp. 2 and the computation of d prime.
• In the Discussion, the authors added the relation to postdiction, the effect of modelling perceptual noise and applying the model to multisensory causal inference (some refs are missing here), and visual motion clustering.*

We have added a few more references to the section on multisensory causal inference, thank you.

“In contrast, work on perceptual causal inference has assumed perceptual noise (Shams & Beierholm 2022, Kording et al 2007).”

*• Basic demographical data cannot be reported because this data was not recorded. I think it is good scientific practice to report at least minimal data on human participants, but for a theoretical contribution this might be less important.*

We apologise, and will ensure it is recorded for future studies.

*• For Exp. 2 , the authors comprehensively justify the imbalance of different/same sequences and why they omitted the confidence ratings.
• The authors do not include a model recovery analysis, arguing that their competing models are only variants of the CRP model. I disagree to that point: Even if the authors use similar models as valid possible alternatives, it still makes sense to check whether model fitting / model analyses pipelines can reliably recover the different models. This is a question of methodological rigor, not a question of whether the competing models are old, new or a special case of the main model (which often holds for simpler competing models). Thus, I still think that model recovery is essential for a modelling paper introducing a novel computational model.*

Following the recommendations of the reviewer, we now include a model recovery section in the supplementary material, replicated here:

“Any attempt at creating a new model faces issues of not just parameter identification and recovery, but also model recovery. A model that (e.g. through error) always assigned a ELBO value of 0 (e.g.) may seem like it is able to explain a lot of data, but is obviously broken. However, this may only be noticable when testing it on data sets where it is known that it should perform poorly (as data were actually generated through a different process).

To ensure that we do not have any issues with the CRP model claiming good model fits on incompatible data, we performed a model recovery exercise.

We repeated the methods above (see Parameter recovery above), but for each of the models CRP, alternative A, alternative B, and alternative C. For each of the four models 45 data sets were created (45 x 4 models = 180 datasets).

Having generated the simulated data we then fitted each of the four models to each of the data sets (4 x 180 model fits). This allowed us to confirm whether the inferred model, after model fitting and comparison, is indeed the generating 'True' model.

Figure 5 shows that while the correct model is generally inferred, occasionally data generated from model CRP (and similarly from model alt B) can be erroneously inferred to be from model alt A. In practice, this means that our model results in the main paper may actually be slightly under estimating the strength of the CRP model.

Figure 5: Confusion matrix for model recovery. True generating model on the horizontal

axis, while the inferred model after model fitting and comparison is on the vertical axis.

In the case of a perfect model recovery the proportion inferred would be zero away from

the diagonal. “

*Reviewer #3: The authors have responded satisfactorily to my comments. However, they have introduced some text that is confusing.

Authors write:
"By default, the model is biased towards the most parsimonious interpretation of events (Occam’s razor: as little different causes as possible)"
Parenthetical is awkward and grammar is off. How about:
"By default the model is biased towards the most parsimonious interpretation of events (Occam's razor), which in this case means creating as few different causes as possible."*

We are grateful for the reviewer’s comments and have followed their suggestion.

*A paragraph starts with the topic sentence "Importantly, the model goes beyond giving just the number of sources, but says which sounds are produced by each source." They have now added sentences to the end of the paragraph about alternative strategies -- I don't see how this fits the paragraph or relates to the previous sentence.*

The sentences were added at suggestion by reviewer 2. It is an ongoing discussion in computational models of perception of how the brain converts a full posterior distribution into a single response. Typical candidates are the max (MAP) or mean of the posterior, or by drawing samples from the posterior. However other strategies can potentially be used.

In this case we agree with reviewer 2 that this is an appropriate placement of these sentences.

*"a very low number of the most plausible (or even a single) interpretations in memory." A model that maintains only one interpretation can't postdict. I think authors should cut "(or even a single)".*

See below

*Paragraph at Line 460: Authors say that THEIR use of simplifications/approximations suggests that the BRAIN, which draws on a larger stream of perceptual data, may use even simpler approximations. This reasoning doesn't seem quite right to me. Isn't it simply the case that ANY system trying to implement this algorithm must either deal with an exploding number of possible interpretations or make substantial simplifications to the algorithm? The authors had to contend with that in their program, and the brain has to contend with that. Unless the authors do not agree with this analysis, I think that is what they should say.*

In accordance with the reviewer’s two comment we have now modified the entire paragraph to say

“While the current implementation improves on the intractability of traditional

parametric models when the number of possible sources exceeds a few, numerical

approximations were still necessary to manage the model’s complexity. This brings the

model closer to mimicking the brain’s constant, automatic, and effortless causal

inference. **Due to the potential complexity of doing inference over a large number of stimuli, and the exploding number of possible interpretations, any computational system will need to employ approximations or simplifications. What approximations or simplifications the nervous system uses is as of yet unclear.**”
